# Initiated Chemical Vapor Deposition of Crosslinked Organic Coatings for Controlling Gentamicin Delivery

**DOI:** 10.3390/pharmaceutics12030213

**Published:** 2020-03-01

**Authors:** Gianfranco Decandia, Fabio Palumbo, Annalisa Treglia, Vincenza Armenise, Pietro Favia, Federico Baruzzi, Katrin Unger, Alberto Perrotta, Anna Maria Coclite

**Affiliations:** 1 Department of Chemistry, University of Bari Aldo Moro, Via Orabona 4, 70126 Bari, Italy; gianfranco.decandia@uniba.it (G.D.); annalisa.treglia@uniba.it (A.T.); vincenza.armenise@uniba.it (V.A.); pietro.favia@uniba.it (P.F.); 2 Institute of Nanotechnology, National Research Council of Italy, c/o Department of Chemistry, University of Bari Aldo Moro, Via Orabona 4, 70126 Bari, Italy; 3 Institute of Sciences of Food Production, National Research Council of Italy, Via G. Amendola 122/O, 70126 Bari, Italy; federico.baruzzi@ispa.cnr.it; 4 Institute of Solid State Physics, NAWI Graz, Graz University of Technology, Petersgasse 16, 8010 Graz, Austria; katrin.unger@tugraz.at (K.U.); alberto.perrotta@gmail.com (A.P.); anna.coclite@tugraz.at (A.M.C.)

**Keywords:** initiated chemical vapour deposition, drug delivery, poly(methyl methacrylate) (PMMA) copolymer, antibacterial test

## Abstract

A coating consisting of a copolymer of methacrylic acid and ethylene glycol dimethacrylate was deposited over a gentamicin film by initiated chemical vapor deposition with the aim of controlling the drug release. Gentamicin release in water was monitored by means of conductance measurements and of UV-vis Fluorescence Spectroscopy. The influence of the polymer chemical composition, specifically of its crosslinking density, has been investigated as a tool to control the swelling behavior of the initiated chemical vapor deposition (iCVD) coating in water, and therefore its ability to release the drug. Agar diffusion test and microbroth dilution assays against Staphylococcus aureus and Pseudomonas aeruginosa on cellulose coated substrates confirmed that the antibacterial activity of the drug released by the coating was retained, though the release of gentamicin was not complete.

## 1. Introduction

Gentamicin is a broad-spectrum aminoglycoside antibiotic produced from *Micromonospora purpurea* and active against a wide range of gram-positive and gram-negative bacteria [1]. It is a mixture of four major congeners designated as C_1_, C_2_, C_1a_ and C_2a_, differing in their degree of methylation on the purpurosamine ring [2]. Gentamicin binds irreversibly to the bacterial 30S ribosomal subunit, causing misreading of t-RNA, leaving the bacterium unable to synthesize proteins vital to its growth [3]. The effectiveness of its antibacterial activity is however undermined by its high water solubility, which hinders the penetration into cells and, in turn, the treatment of intracellular bacterial infections. Therefore, several studies are nowadays devoted to the development of controlled delivery systems that could address the bioactive agents to the site of infection and maintain a high local concentration without producing adverse systemic effects due to prolonged exposure to the antibiotic.

Drug delivery systems are engineered technologies for the controlled release or targeted delivery of therapeutic agents from a variety of polymeric and inorganic vehicles. Several synthetic approaches based on immobilization or in situ release of bactericidal substances such as antibiotics and metal derivatives have been extensively explored to produce antibacterial layers [4,5,6,7]. In situ delivery of antimicrobials can be achieved embedding the active compounds into polymeric matrices; these latter thus act as barriers limiting their diffusion in the surrounding medium. The release kinetics can be controlled by properly choosing the characteristics of the polymeric matrix: normally a hydrophilic behavior of the polymer can speed the release rate up, letting water penetrate easily in the polymeric network of the film and favoring the leakage of the active additive [8].

A fine level of control on drug release kinetics can be acquired when hydrogel coatings are employed (for example, from poly(ethylene glycol) di-acrylate and its co-polymers): hydrogels, in fact, can swell when in contact with an aqueous environment because of massive water absorption. This makes polymeric network looser and creates larger paths for the diffusion of the active compound [9,10,11]. In addition, it is also possible to synthesize stimuli-responsive hydrogels, which are able to release drug upon a specific stimulus, such as changes in pH or temperature [12,13,14,15,16]. Poly(methacrylic acid) (PMAA) nanogels exhibit a sharp volume phase change upon increasing pH: in acid conditions, the carboxylic groups are not ionized, and the PMAA nanogels are in a collapsed state, whereas at higher pH values, the coulombic repulsions between the deprotonated carboxylic groups induce the swelling of the nanogels [12]. These systems are helpful to protect sensitive drugs from the acid environment of the stomach or in cases where a specific release in the gastrointestinal tract is required.

Stimuli-responsive hydrogels can be successfully deposited by initiated chemical vapor deposition (iCVD): a low-energy, one step, film forming process that can be advantageously used to embed a bio-active molecule in a polymer encapsulant layer. This technique allows for solvent-free processing under mild conditions, thus minimizing a potential impact on the integrity of the active compound, and retention of the monomer structure in the encapsulating coating [17,18].

Examples of drug delivery systems based on iCVD are present in literature. Ibuprofen [19] and campothecin [20] were encapsulated within a crosslinked PMAA thin film deposited by means of iCVD. In both cases, the drug release was pH dependent: at low pH the cross-linked film acted as diffusion barrier, while at neutral pH the film allowed the free diffusion of the drug out of the encapsulant. The crosslinker density in the polymer coating allowed to tune the release kinetics of several orders of magnitude, from hours to days [21]. In another study, McInnes et al. [22] reported the iCVD deposition of a temperature-responsive polymeric coating of poly(*N*-isopropylacrylamide-*co*-diethylene glycol divinyl ether) (pNIPAM-*co*-DEGDVE) onto porous silicon loaded with an anticancer drug. Thanks to the thermoresponsive nature of such layer, the drug release was temperature dependent. Werzer et al. expanded such study exploring the combined effect of pH and temperature on different drugs delivered from pNIPAM-*co*-DEGDVE encapsulants [23]. This study highlighted that pH and temperature influenced both drug delivery rate and its interaction with the barrier coating.

In this work, a thin film of gentamicin has been encapsulated by means of iCVD with a methacrylic acid (MAA) based film with a variable crosslinking degree, modulated by the addition of ethylene glycol dimethacrylate (EGDMA). Previous works were mostly based on model drugs, of which relatively uniform thin films are easily accessible from simple solution processes such as spin coating or drop casting from organic solvents that easily wetted the glass substrates. Thin films of water-soluble drugs, like gentamicin, are not obtained easily on glass. In this regard, the present work advances the state of art by demonstrating the encapsulation and controlled release of a water-soluble antibiotic.

## 2. Materials and Methods

### 2.1. Materials

Methacrylic acid (99%, Merck, Darmstadt, Germany), ethylene glycol dimethacrylate (98%, Merck, Darmstadt, Germany) and tert-butyl peroxide (TPBO, 98%, Merck, Darmstadt, Germany) were used in the iCVD process as, respectively, monomer, crosslinker and initiator without further purification. The drug layer was obtained from solutions of Gentamicin Sulphate (powder, from Apollo Scientific) in distilled water.

Square silicon shards (2.5 × 2.5 cm^2^) were used as substrates for Fourier Transform Infrared (FTIR), and ellipsometry analyses. Rectangular silicon shards (2.5 × 5 cm^2^) were used for swelling experiments. Conventional glass slides (cut into 2.5 × 2.5 cm^2^ squares) were used as substrates for conductimetry release tests.

### 2.2. Deposition of the Drug Layer

The drug thin film was obtained by drop casting and spin coating on silicon and glass substrates treated with an O_2_ glow discharge (Femto, version 1, Diener Electronic) for 2 min at 100 W/cm^2^ and 100 mTorr, to generate a more hydrophilic surface and to improve the adhesion of the layer.

Drop casted layers were prepared by placing 20–500 µL of gentamicin solution (10 mg/mL) onto carefully leveled substrates. Samples were then stored at room temperature for 24 h under a fume hood to let the solvent evaporate.

Spin coated layers were prepared using a standard spin coating device (Ingenieurbüro Reinmuth, Germany): 300 µL of gentamicin solution (100 mg/mL) were dropped onto the substrates and spun between 1000–2000 rpm. The spin time was set to 50 s, to complete the solvent evaporation.

### 2.3. Initiated Chemical Vapor Deposition Reactor Setup

iCVD layers were deposited using a custom-built setup, as described in ref. 21. Substrates were placed in a cylindrical vacuum chamber, on a stage thermoregulated at 30 °C by a water recirculating chiller.

The MAA and EGDMA monomers were heated to 70 °C and 75 °C, respectively, and fed into the reactor through a heated mixing line. The vapor flow rate was controlled by needle valves. The initiator, TBPO, was kept at room temperature and fed into the reactor through a mass flow controller. The flow-rate of MAA (3–5 sccm) and EGDMA (0.07–0.2 sccm) was varied, to obtain polymers with different degree of crosslinking, while the flow rate of TBPO was fixed at 0.8 sccm. The deposition was carried out at a constant working pressure of 500 mTorr, and at a filament temperature of 300 °C. The polymer layers were grown up to a thickness of 200 nm, monitored in situ by laser interferometry.

### 2.4. Samples Characterization

The thickness of the drug layer was measured by means of a Dektak IIA profilometer.

FT-IR was carried out to determine the chemical composition of the iCVD coatings. FT-IR spectra (4 cm^−1^ resolution, in the range 800–4000 cm^−1^) were obtained in transmission mode with a Bruker IFS 66 v/s spectrometer. Data were processed with OriginPro and a software developed by M. Tazreiter et al. [24], allowed for the determination of crosslinker content in the coatings.

Ellipsometry (M-2000V, J.A. Woollam Co. Inc., Lincoln, NE, USA) was performed primarily to measure the thickness of the iCVD coating, collecting data at three incidence angles (65°, 70° and 75°) in the 370–1000 nm wavelength range. Experimental data were fitted with CompleateEASE^®^ software (J.A. Woollam Co. Inc., Lincoln, NE, USA). The iCVD polymer was modeled with a Cauchy function since it is transparent in the measured wavelength range. To evaluate the swelling behavior of the coating, the samples were placed in a liquid cell (.A. Woollam Co. Inc., Lincoln, NE, USA), filled with distilled water and ellipsometer measurements were collected at a single incidence angle of 75°. The effective medium approximation (EMA) was used to model the film as a composite consisting of polymer and water. The model mixes the optical constants of water with those of the dry Cauchy layer (i.e., polymer) according to their relative fraction (the fitting parameter).

### 2.5. Gentamicin Release Tests

To evaluate gentamicin release through the iCVD coatings, Electrochemical Impedance Spectroscopy (Gamry Instrument, Reference 600) has been used in “single frequency” mode. Since the antibiotic is a sulphate salt, its presence can increase the conductivity, when dissolved in pure water: by monitoring the conductivity variation of the release solution, the gentamicin concentration has been calculated using a calibration curve.

The analysis was carried out in a four-probe configuration, at an AC voltage of 15 mV and at a frequency of 1 kHz. The release test was carried out placing a four electrodes probe kit into 200 mL of distilled water, and, after two minutes from the start of the measurement, the sample was inserted. The release solution was kept at 37 °C and under stirring. Current and voltage were measured every 20 s for 2 h giving the solution impedance as output. The measurements were repeated three times on different samples deposited in the same conditions. Distilled water instead of buffer solutions or simulated body fluids was used to avoid interferences between solution electrolytes and the increase in conductance derived from the dissolution of gentamicin.

The calibration curve was obtained from pure distilled water and addition of 150 µL of gentamicin solution (500 µg/mL) up to cover the desired concentration range. The impedance was measured after every addition.

### 2.6. Antimicrobial Activity Evaluation.

The antimicrobial activity of the gentamicin released by the coatings was evaluated against *Staphylococcus aureus* DSM799 and *Pseudomonas aeruginosa* DSM939 (Leibniz Institute, DSMZ-German Collection of Microorganisms and Cell Cultures GmbH, Braunschweig, Germany).

Immediately after overnight incubation [25], about 10^5^ microbial cells of target strains, handled and standardized as previously reported [26], were plated onto Plate Count Agar (Biolife Italiana srl, Milan, Italy) before disk diffusion assays.

Antimicrobial disk susceptibility assays were carried out as defined by CLSI with slight modification. Positive control filter paper disks (6 mm) charged with 10 µg gentamicin, defined as the breakpoint amount able to distinguish sensitive or resistant strains (http://www.eucast.org/clinical_breakpoints/), as well as sterile filter paper blank disks (6 mm) were purchased from KAIROSafe srl (Trieste, Italy). Standard disks and coated with the encapsulated gentamicin were placed over the inoculated agar surface; after 2 h at 4 °C, plates were incubated at 37 °C for 24 h. Three replicates for each disk were placed onto three different agar plates. Antimicrobial activity was evaluated measuring the area of inhibition zones instead of their diameter.

A six-point standard curve for both *S. aureus* DSM799 and *P. aeruginosa* DSM939 of active gentamicin loaded on blank disk was drawn in the range 20.62–0.64 µg/disk.

In order to compare halos produced over different agar plates, jpg format pictures were imported into Adobe Photoshop CS2 image analysis software (Adobe Systems Incorporated, San Jose, CA, USA); the number of pixel of each halo (excluding the number of pixel relative to the disk) was converted to square millimeter after normalization with the average number of pixel of all disks measuring ~30 mm^2^ [27].

The amount of active gentamicin released from coated disks was then normalized using commercial disks loaded with 10 µg gentamicin. Halo measurements were independently performed at least three times; the amounts of estimated gentamicin released into the agar matrix producing inhibition halos were analyzed using an unpaired two sample equal variance t-test (Microsoft Excel® 2010, after installation of Data Analysis add-in). Difference between the control and treated samples was considered significant at *p* < 0.01.

On the basis of results from disk diffusion assays a further evaluation of gentamicin antimicrobial activity was carried out. Two coated disks were stirred at 37 °C for 2 h in 15 mL of saline solution in order to obtain a 30 µg/mL gentamicin solution. Bacterial cultures, with an average of OD_600nm_ of 0.325 ± 0.05, measured immediately after overnight incubation and corresponding to about 8 log cfu/mL, were diluted 100 times in 180 µL of Oxoid™ Iso-Sensitest Broth (Thermo Fisher Scientific, Milan, Italy) and then 20 µL gentamicin extracted solutions were added, following a two-fold dilution scheme. Bacterial growth was monitored by measuring optical density every 10 min with the Varioskan Flash spectrofluorimeter (ThermoFischer Scientific, Waltham, MA, USA) at a wavelength of 600 nm as previously reported for the evaluation of the effect of antimicrobial activity on microbial growth kinetics [28]. After determination of minimal inhibitory concentration (MIC), the gentamicin concentration at which any increase in OD reading was observed during 24 h of incubation, 20 mL of Iso-Sensitest Broth were inoculated with 200 µL of broth from well of incubated microplate. The minimal bactericidal concentration (MBC) was defined as the lowest concentration at which no growth was observed after incubation (37 °C for 24 h). The assay was replicated independently two times.

To confirm the antibacterial test results, release solutions were analyzed with fluorescence spectrophotometry upon derivatization with o-phtaldehyde (OPA). OPA (40 mg) was dissolved in 1 mL methanol and mixed with 5 mL of sodium tetraborate solution (0.1 M). Then, 50 µL of 2-mercaptoethanol were added and mixed for 1 min. 250 µL of such OPA solution were vigorously mixed with the release aliquot for 1 min and transferred in a cuvette. The analysis was carried out with a fluorescence spectrophotometer (Varian Cary Eclipse; excitation 335 nm, emission 440 nm). Calibration was carried out with gentamicin standard solutions in the range 1–50 µg/mL.

## 3. Results and Discussion

### 3.1. Characterization of the iCVD Coatings on Bare Substrates

**Figure 1:**
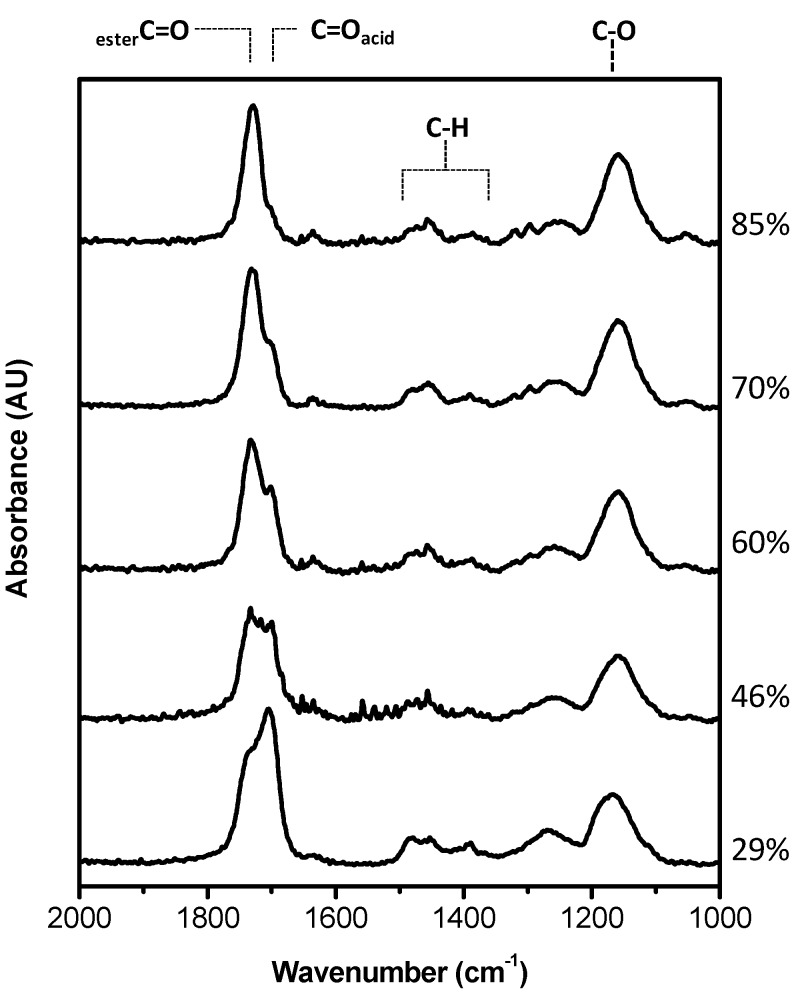
Fourier Transform Infrared (FT-IR) spectra of different copolymers with different methacrylic acid (MAA)/ ethylene glycol dimethacrylate (EGDMA) composition. Percentages on the right are EGDMA film volume fractions.

MAA-EGDMA copolymers were deposited by iCVD varying the fraction of the crosslinker, EGDMA. The chemical analysis of the different copolymers measured by FT-IR is shown in Figure 1 in the range 1000–2000 cm^−1^ and in Supplementary Materials Figure S1 in SI on a larger wavenumber range. The copolymers are characterized by the O–H stretching band relative to the acid group of MAA at 3250 cm^−1^, the carbonyl stretching band, due to carboxylic acid and ester moieties, in the range 1700–1800 cm^−1^, and the C–O band at 1150 cm^−1^ attributable to both monomers structure. In Figure 1, it can be appreciated that the carboxylic C=O absorption is composed of two contributions, one at 1703 cm^−1^ and the other at 1730 cm^−1^, whose relative intensity changes with the composition of the coating. The former is due to the acid carboxylic groups, while the latter component can be attributed to ester moieties.

Starting from the spectra of the MAA and EGDMA homopolymers a best fitting procedure of the FTIR spectra has been carried out to get the composition of the deposited copolymers as a percentage of each monomer, and in turn the crosslinker volume fraction, as it can be observed in Figure S2 in Supplementary. The copolymer spectrum was fitted with a linear combination of the corresponding homopolymer absorbance spectra, following the routine explained in ref. [24] The evaluated composition in terms of crosslinker percentage is reported in Figure 1.

**Figure 2:**
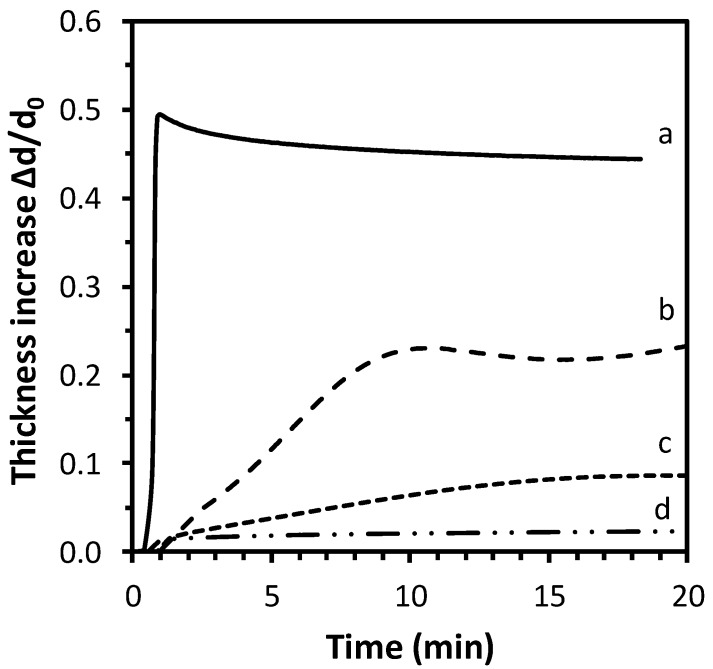
Swelling behavior, in terms of thickness increase, Δd/d_0_, of different iCVD coatings with 29% (a), 60% (b), 70% (c) and 85% (d) EGDMA. The error bar in the measurements is ±1%.

Since the coatings should work as drug-release systems in an aqueous environment, the swelling in water was tested depending on the crosslinking. The swelling behavior is very important since it is correlated to the drug diffusion through the iCVD polymer and then to the release kinetics [29]. In Figure 2 it can be observed that the thickness of different iCVD coatings increases due to the swelling in water, as measured by means of ellipsometry: the coating with the lowest fraction of EGDMA (29%) swells the most, with around 45% of water uptake (in terms of thickness increase), while the one with 85% of EGDMA shows negligible swelling. Polymers containing less than 29% of EGDMA dissolved in water. The amount of crosslinker leads to changes in the mesh size (i.e., the distance between two consecutive crosslinks) of the polymers and this in turn affects the diffusivity of the water in/out of the layer [21]. In particular, a higher amount of crosslinker leads to a smaller mesh and less swelling, as it can be seen in Figure 2. The small thickness loss for the 29% EGDMA sample, observed after 1 min immersion, can be related to a mass loss. It is common for iCVD hydrogels, characterized by important swelling, to lose some weakly bond oligomers present on the surface at the immersion in water. The sample with 60% EGDMA shows a bump in the thickness increase curve that could be considered within the experimental error.

### 3.2. Gentamicin Release Tests

Drug delivery systems were prepared by depositing the iCVD MAA-EGDMA copolymers on top of a thin film of gentamicin. The first step was the optimization of the deposition of a homogeneous gentamicin thin film. As it can be observed in the supporting information file (Table S1 in SI), drop casted gentamicin resulted very non homogeneous in thickness: the difference in thickness between the center and the border was very important. The thickness of the spin coated coatings was more homogeneous (thickness standard deviation in thickness around 5%) and controllable by changing the speed rotation rate and spin solution concentration.

**Figure 3:**
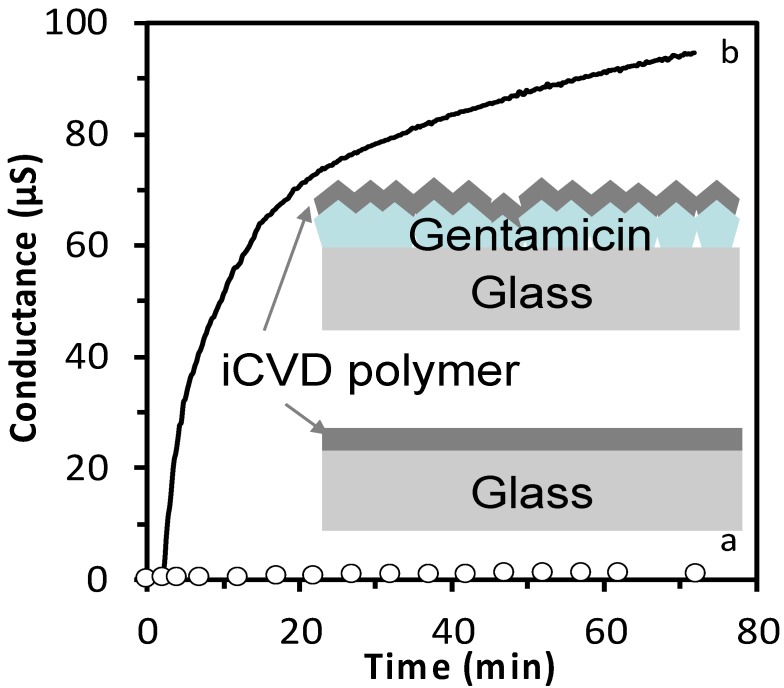
Conductance variation due to the release test from (a) initiated chemical vapor deposition (iCVD) coating alone (white dots) and (b) drug layer with iCVD coating on top (continuous line). Drug layer obtained by drop casting (drop volume = 300 µL, [Gent] = 10 mg/mL), iCVD coating thickness ~200 nm with an EGDMA volume fraction of 46%, immersed in distilled water.

The gentamicin release was assessed by measuring the conductance of the released solution. Before investigating the drug release, the variation of conductance due to drug-free iCVD polymer in solution was evaluated. Figure 3 shows a comparison between the conductance of a drug containing layer (300 µg gentamicin solution 10 mg/mL, drop casted) with a iCVD polymer (200 nm thick, 46% EGDMA) on top and the same iCVD polymer without gentamicin. The sample containing gentamicin, once dipped in pure water, lead to a variation of conductance of almost 100 µS in 72 min, differently from the gentamycin free iCVD polymer that gave negligible conductance variation. This result indicated that the iCVD coating does not influence the detection of the drug.

**Figure 4:**
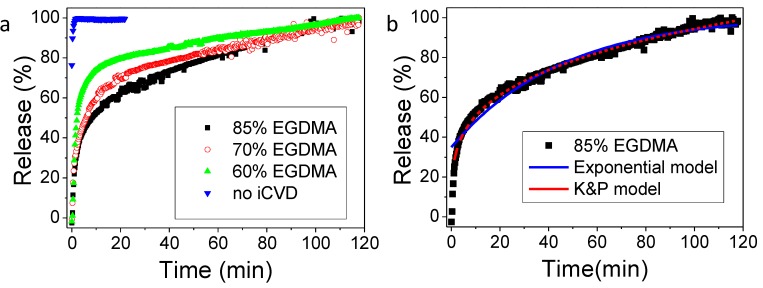
(**a**) Percentage of released gentamicin from spin coated samples ([Gent] = 100 mg/mL, v = 1500 rpm) without iCVD coating and coated with a 200 nm iCVD layer with different EGDMA volume fractions: 60%, 70% and 85%. Average values obtained measuring the conductivity of three replicates, a maximum standard deviation of 12% was estimated for each data point. Total content of gentamicin is 45 ± 5 µg/cm^2^. (**b**) Gentamicin release data for the 85% EGDMA polymer coating fitted with the exponential model of Equation (1) and with the Korsmeyer and Peppas model.

Once the calibration was accomplished, as reported in Figure S3, the gentamicin release was investigated. In Figure 4 the results for the sample obtained from drug spin coating (1500 rpm, 100 mg/mL gentamicin) and iCVD film deposition with different percentages of crosslinker, together with an uncovered gentamicin spin coated layer, are reported. It can be observed that the addition of the iCVD layer effectively limits the drug release. In particular, the higher the crosslinker content, the slower the gentamicin release rate is. Higher release rates can be related also to the larger swelling of the samples with lower crosslinking degree.

More details on the release mechanism can be understood by the mathematical models applicable to the release data. Previously, good fits were obtained by applying the diffusion model based on reservoir systems, in which the drug is encapsulated by an outer membrane and gradually released [21,23]. In such model, the rate limiting step is the diffusive transport of the drug through the membrane, and therefore the release can be described by an analytic expression derived by the Fick’s law, which works under the conditions that the drug is released in a volume of solution at least three times higher than the volume required for a saturated solution (i.e., sink condition), that there is a negligible (or rapid initial) polymer swelling and that the drug permeability does not change in time. This model describes the time-dependence of the fractional release *M_t_*/*M*_∞_ as:

(1) $$\frac{M_{t}}{M_{\infty }}=M_{max}\left(1-e^{-k_{er}\left(t-t_{0}\right)}\right)$$

where *k_er_* is an effective release constant, *M_max_* estimates the maximum amount of drug release from the system under investigation and t_0_ is a lag time, accounting for a dissolution onset different from the start of the experiment. t_0_ was added as fitting parameter to take into account that since this release study concerns very crosslinked films, the hypothesis of a negligible or rapid initial swelling may not hold. The fitting results can be observed in Figure 4b for the 80% EGDMA and Table 1. The R^2^ values are close to unity for most of the systems, indicating a good agreement of the model with the experimental data. The release constant, *k_er_*, decreases from the uncoated sample to the coated ones and among those, the one with the 85% EGDMA shows the lowest release constant. The values for the release constants obtained in this study are in agreement with the release constants obtained by previous studies based on similar crosslinked hydrogels [21].

Another simple model that is usually applicable to such thin film systems for drug release is the Korsmeyer and Peppas model, even though it mostly describes matrix systems, where the drug is embedded in the polymer and not coated by it, like in reservoir systems. Nevertheless, the Korsmeyer and Peppas model could also fit our experimental data with R^2^ values closer to unity than the exponential model. The coefficient in the Korsmeyer and Peppas model can give hints on the different types of transport mechanisms. [29] Coefficients below 0.5, like those obtained with our fittings (Table 1), are characteristics of Fickian transport. The Korsmeyer and Peppas model was not applicable to the experimental release data from the bare drug, i.e., without iCVD coating.

For completeness, when gentamicin was deposited by drop casting the barrier effect of the iCVD coating was not effective, likely because of the non-homogeneity of the active layer, as shown in Figure S4 in Supplementary. Optical microscope images have shown, in fact, defects on the iCVD coatings deposited onto drop casted gentamicin: these may have negatively influenced the barrier characteristics of the iCVD polymer. The defects are present before immersion in water and they are attributable to the fact that the underlaying layer of gentamicin is so rough that it is difficult to obtain a continuous, low-defective iCVD layer on top, in the chosen deposition conditions.

### 3.3. Antibacterial Activity of the Samples

In order to compare the antimicrobial activity of 30 µg gentamicin loaded cellulose disks coated with different iCVD polymers, a regression curve of gentamicin antimicrobial activity was drawn for *S. aureus* DSM799 and *P. aeruginosa* DSM939. On the basis of these results it was possible to estimate the amount of active gentamicin released by the disks as reported in Table 2.

For both pathogens, the estimated amount of active gentamicin released from 60% EGDMA coated disks, calculated by using the regression curve of antimicrobial activity (area of inhibition halos) produced by a gentamicin solution, resulted to be significantly different (*p* < 0.01) from 70% and 85% EGDMA coated disks, according to independent t-test evaluation.

**Figure 5:**
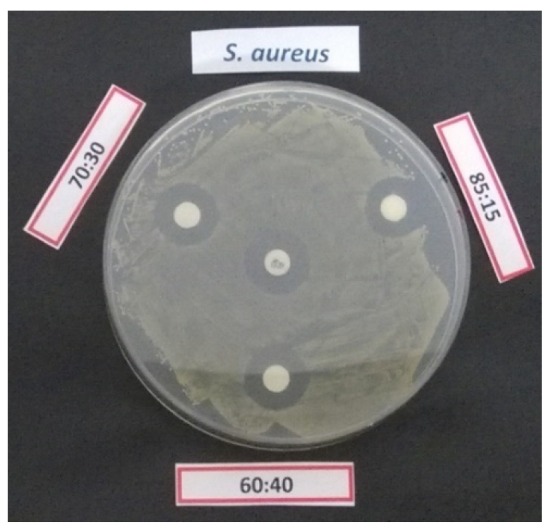
Inhibition halos produced by gentamicin against *S. aureus* DSM799 released by cellulose disks coated with different iCVD polymers of different crosslinker content (as reported in the labels). The positive disk control (10 µg gentamicin/disk) is in the center.

An example of inhibition halo produced by gentamicin loaded cellulose disks coated with different iCVD polymer against *S. aureus* DSM799 is presented in Figure 5.

After 24 h incubation, it can be observed that the samples released less than 30 µg active gentamicin, the amount originally loaded. In addition, these results confirmed that the gentamicin release through the less crosslinked coating (60% EDGMA) is more important, as demonstrated by the higher activity by microbroth dilution method.

In order to understand if the reduced activity found in disk samples could be due to limitation in the release or in partial inactivation of gentamicin, samples were kept in physiologic solution as described in “material and methods” section and surnatant were tested for antibacterial activity.

On the basis of 24 h growth kinetic it was possible to define MIC and, after a following incubation in fresh microbiological medium, the MBC values, in the hypothesis that gentamicin for each disk was completely released and totally active (maximum 20 µg/mL in broth). The estimated amount gentamicin producing MIC and MBC from the disk coated with 60% EDGMA iCVD polymer, compared to that obtained by gentamicin stock solution is shown in Table 3.

Apparently, the measured activity was about half the one expected for the dropped gentamicin amount. To better rationalize this result, the amount of gentamicin in the extracted solutions was determined by fluorescence spectroscopy upon OPA derivatization. This analysis led to an amount of gentamicin released from the iCVD coated samples equal to 18 ± 1 µg, instead of expected 30 µg which means that the MIC and MBC values were overestimated. Considering the actual released gentamicin concentration, i.e., 60% of the estimated one, it is possible to correct the MIC value, obtaining 3 µg/mL. This result indicates that the gentamicin is not completely released from the cellulose disc, but the part released is more or less as active as the standard gentamicin, although the microdilution method is unable to define the real MIC value of coated cellulose disk, as a single value between 2.5 µg/mL and 5 µg/mL.

The reason for the reduced release could be related to a strong coupling of the iCVD coating to the cellulose membrane resulting in a more difficult release of the coated drug. This topic deserves further studies to optimize substrate and coating coupling.

## 4. Conclusions

Different drug release behaviors were achieved by depositing an iCVD polymer onto a gentamicin layer, deposited by different techniques. The release kinetic strongly depends on the chemical composition of the iCVD polymers: in particular, it has been shown that gentamicin release slowed down when the EGDMA volume fraction of the coatings was increased. An increase of the volume fraction of the EGDMA from the 70% to the 85% reduces the drug released after twenty minutes of twenty percentage points. This is due to the increased crosslinking as a consequence of the presence of EGDMA, that alter the swelling characteristics of the barrier coating. In addition, it has been demonstrated that the way the gentamicin is deposited affects the homogeneity of the drug layer and in turn of the iCVD polymer and the antibiotic release as well. Samples obtained by spin coating and drop casting coated with an iCVD layer containing 85% of EGDMA released in water respectively the 70% and the 90% of the loaded gentamicin after 40 min.

As far as antimicrobial activity is concerned, it can be concluded that released gentamicin retain most of the antibacterial activity, demonstrating that such process does not induce alteration in the antibiotic molecule. However, for reasons that necessitate more investigations, but likely associated to the coupling of the barrier coating with the three-dimensional support, about 40% of coated gentamicin is not released from the cellulose disks used to determine the gentamicin activity.

## Figures and Tables

**Table 1:** Fitting results of the release data. The first four parameters refer to the model of Equation (1). The last two to the Korsmeyer and Peppas model.

Sample	R^2^	*M_max_*	*k_er_* (min^−1^)	*t*_0_ (min)	R^2^ (K & P model)	N (K & P model)
No iCVD	0.99	99.5	2.86	0.1	-	-
EGDMA 60%	0.81	99.3	0.06	11.9	0.91	0.14
EGDMA 70%	0.92	90.0	0.04	11.4	0.97	0.21
EGDMA 80%	0.92	93.7	0.02	11.8	0.99	0.27

**Table 2:** Amount of active gentamicin released from gentamicin loaded disks against the pathogens.

Bacteria	EGDMA Volume Fraction		
	60%	70%	85%
*S. aureus* DSM799	12.0 ± 0.5 µg	7.1 ± 0.4 µg	8.0 ± 0.8 µg
*P. aeruginosa* DSM939	13.1 ± 0.3 µg	7.9 ± 0.2 µg	8.9 ± 0.7 µg

**Table 3:** Minimal inhibitory concentration (MIC) and minimal bactericidal concentration (MBC) values, in µg/mL, of active gentamicin released from disks producing MIC in comparison with that produced by free gentamicin in water. iCVD layer prepared with an EGDMA volume fraction of 60%.

Extracted solution	*S. aureus* DSM799		*P aeruginosa* DSM939	
	MIC	MBC	MIC	MBC
Gentamicin released from iCVD coated disks	5	10	5	10
Gentamicin control solution	2.5	5	2.5	5

## Supplementary Materials

The following are available online at https://www.mdpi.com/1999-4923/12/3/213/s1, Figure S1: FTIR spectrum of iCVD MAA/EGDMA copolymer with 46% crosslinker, Figure S2: Example of the best fitting of the infrared spectrum of an iCVD MAA-EGDMA (85%) copolymer Table S1: Thickness of drug layer deposited by drop casting or spin coating, Figure S3. Calibration curve of electrochemical impedance spectroscopy (EIS) conductivity measurement of gentamicin, and corresponding fitting, Figure S4. Gentamicin release from drop casted samples.
