# Supplementary material for: Initiated Chemical Vapor Deposition of Crosslinked Organic Coatings for Controlling Gentamicin Delivery
Source: Pharmaceutics. 2020 Mar 1;12(3):213. doi: 10.3390/pharmaceutics12030213 (PMC7150873; doi:10.3390/pharmaceutics12030213)
Supplement: Supplementary file 1 [file pharmaceutics-12-00213-s001.pdf]

pharmaceutics

Supplementary Materials: Initiated Chemical Vapor Deposition of Crosslinked Organic Coatings for Controlling Gentamicin Delivery

Gianfranco Decandia [1], Fabio Palumbo [2,*], Annalisa Treglia [1], Vincenza Armenise [1],
Pietro Favia [1,2], Federico Baruzzi [3], Katrin Unger [4], Alberto Perrotta [4] and Anna Maria Coclite [4]

1. Infrared Absorption Spectroscopy of Initiated Chemical Vapor Deposition (iCVD) Coating

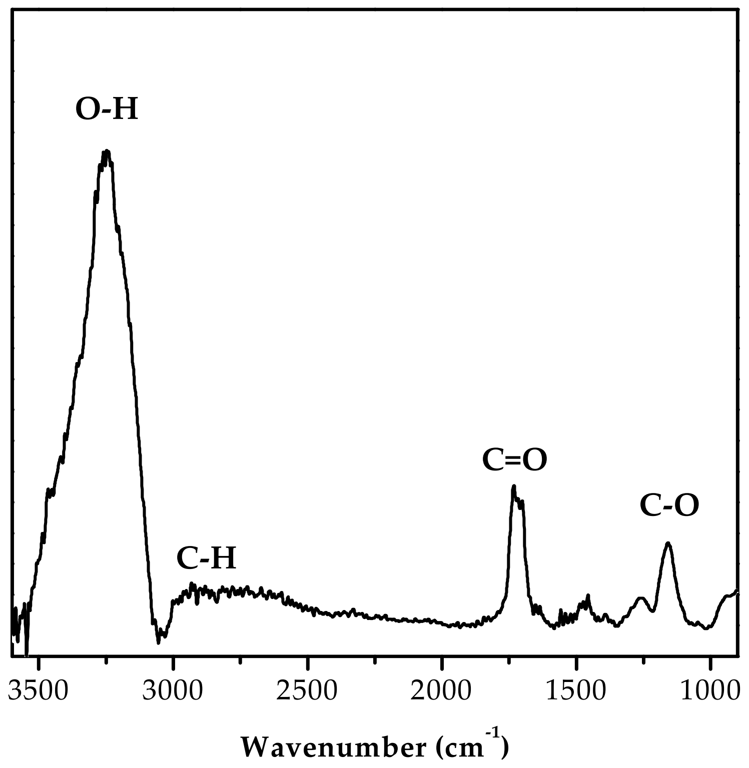

Figure S1. FTIR spectrum of initiated chemical vapor deposition (iCVD) methacrylic acid/ethylene glycol dimethacrylate (MAA/EGDMA) copolymer with 46% crosslinker.

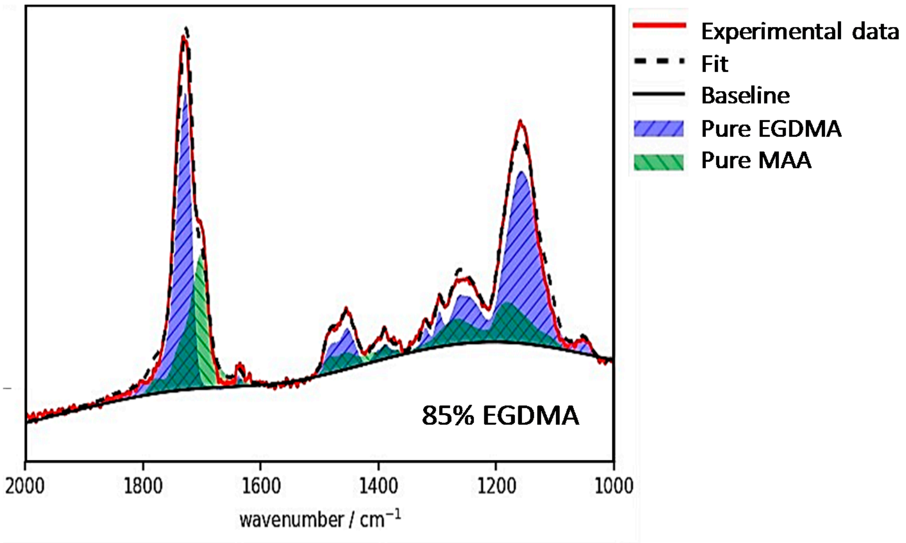

Figure S2. Example of the best fitting of the infrared spectrum of an iCVD MAA-EGDMA (85%) copolymer.

2. Active Layer Thickness.

In Table S1 the thickness measurement of the drug layer is reported for gentamicin deposited onto polished silicon by drop casting, at different solution concentration, and spin coating, from a 100mg/mL solution). It can be seen that the coating is very inhomogeneous in thickness when deposited from drop casting, resulting much thicker in the border.

Table S1. Thickness of drug layer deposited by drop casting or spin coating.

Sample	Centre	Borders
Drop Casting 200 µL (10 mg/mL)	0.9 ± 0.3 µm	1.9 ± 0.9 µm
Drop Casting 300 µL (10 mg/mL)	1.5 ± 0.1 µm	4.0 ± 1.2 µm
Spin Coating 1500 rpm (50 mg/mL)	110 ± 6 nm	
Spin Coating 1000 rpm (100 mg/mL)	260 ± 15 nm	
Spin Coating 1500 rpm (100 mg/mL)	210 ± 10 nm	
Spin Coating 2000 rpm (100 mg/mL)	180 ± 10 nm	

3. Gentamicin Release.

In Figure S3 the results of calibration of the four probe electrochemical impedance spectroscopy conductivity measurement are reported.

The calibration has been obtained by addition of successive aliquots of known content of gentamicin.

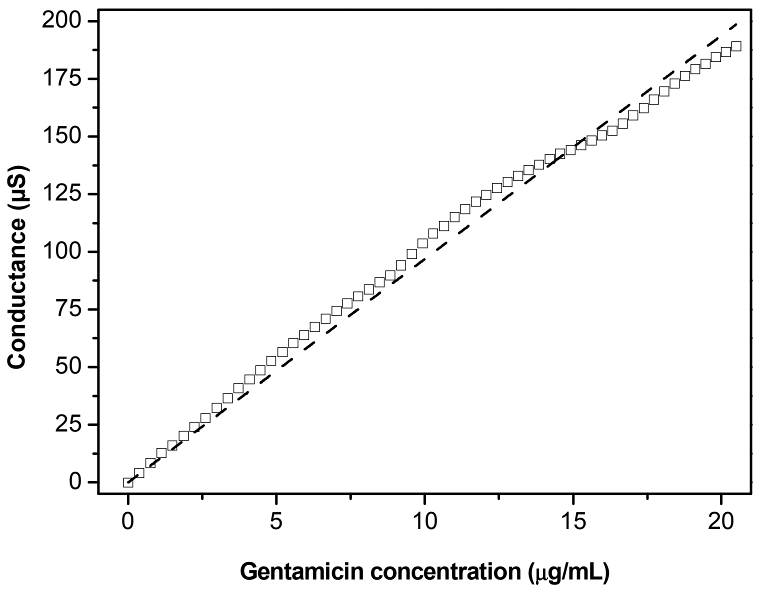

Figure S3. Calibration curve of EIS conductivity measurement of gentamicin, and corresponding fitting result.

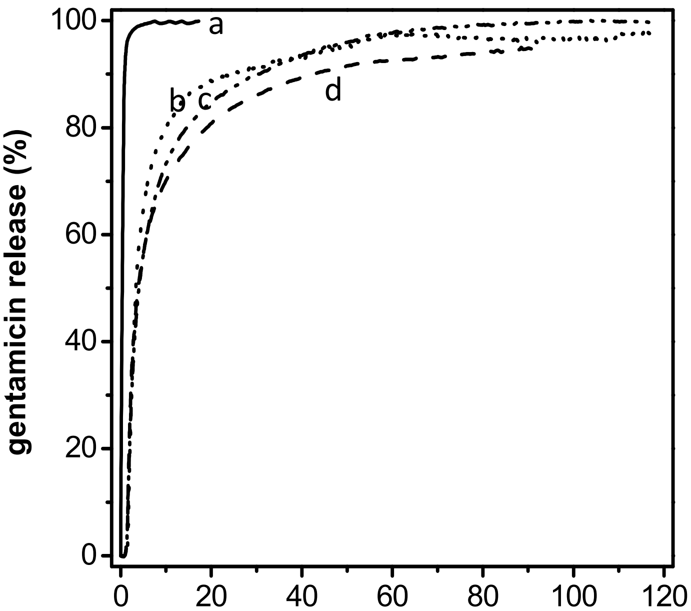

Figure S4. Gentamicin release from drop casted samples (drop volume = 300 µL, [Gent] = 10 mg/mL) without iCVD coating (a) and coated with a 200 nm iCVD layer with different EGDMA volume fractions: (b) 60% (c) 70% and (d) 85%. Total content of gentamicin is 480 ± 14 µg/cm^2.
